# Fabrication of naturel pumice/hydroxyapatite composite for biomedical engineering

**DOI:** 10.1186/s12938-016-0203-0

**Published:** 2016-07-07

**Authors:** Baran Komur, Tim Lohse, Hatice Merve Can, Gulnar Khalilova, Zeynep Nur Geçimli, Mehmet Onur Aydoğdu, Cevriye Kalkandelen, George E. Stan, Yesim Muge Sahin, Ahmed Zeki Sengil, Mediha Suleymanoglu, Serap Erdem Kuruca, Faik Nuzhet Oktar, Serdar Salman, Nazmi Ekren, Anton Ficai, Oguzhan Gunduz

**Affiliations:** Orthopaedics and Traumatology Department, Kanuni Sultan Suleyman Training and Research Hospital, Kucukcekmece, Halkali, 34303 Istanbul, Turkey; Faculty of Engineering, Institute for Materials Science, Christian-Albrechts-University Kiel, 24143 Kiel, Germany; Department of Bioengineering, Faculty of Engineering, Marmara University, Istanbul, Turkey; Department of Pharmaceutical Biotechnology, Institute of Health Sciences, Marmara University, Istanbul, Turkey; Department of Industrial Product Design, Bachelor Science, Istanbul Arel University, Istanbul, Turkey; Department of Biology, Bachelor Science, Faculty of Arts and Sciences, Marmara University, Istanbul, Turkey; Vocational School of Technical Sciences, Biomedical Devices Technology Department, Istanbul University, Istanbul, Turkey; National Institute of Materials Physics, 077125 Magurele-Ilfov, Romania; Department of Biomedical Engineering, Faculty of Engineering–Architecture, Istanbul Arel University, Istanbul, Turkey; School of Medicine, Department of Medical Microbiology, Medipol University, Istanbul, Turkey; Department of Physiology Istanbul Medical Faculty, Istanbul University, Istanbul, Turkey; Advanced Nanomaterials Research Laboratory, Department of Metallurgy and Materials Engineering, Faculty of Technology, Marmara University, Goztepe Campus, 34722 Istanbul, Turkey; Department of Metallurgy and Materials Engineering, Faculty of Technology, Marmara University, Goztepe Campus, 34722 Istanbul, Turkey; Department of Electrical and Electronics Engineering, Faculty of Technology, Marmara University, Istanbul, Turkey; Faculty of Applied Chemistry and Materials Science, University Politehnica of Bucharest, 1-7 Polizu Street, 011061 Bucharest, Romania

**Keywords:** Bioceramics, Natural pumice, Bovine hydroxyapatite, Bioinspired composites

## Abstract

**Background:**

We evaluated the Bovine hydroxyapatite (BHA) structure. BHA powder was admixed with 5 and 10 wt% natural pumice (NP). Compression strength, Vickers micro hardness, Fourier transform infrared spectroscopy, scanning electron microscopy (SEM) and X-ray diffraction studies were performed on the final NP-BHA composite products. The cells proliferation was investigated by MTT assay and SEM. Furthermore, the antimicrobial activity of NP-BHA samples was interrogated.

**Results:**

Variances in the sintering temperature (for 5 wt% NP composites) between 1000 and 1300 °C, reveal about 700 % increase in the microhardness (~100 and 775 HV, respectively). Composites prepared at 1300 °C demonstrate the greatest compression strength with comparable result for 5 wt% NP content (87 MPa), which are significantly better than those for 10 wt% and those that do not include any NP (below 60 MPa, respectively).

**Conclusion:**

The results suggested the optimal parameters for the preparation of NP-BHA composites with increased mechanical properties and biocompatibility. Changes in micro-hardness and compression strength can be tailored by the tuning the NP concentration and sintering temperature. NP-BHA composites have demonstrated a remarkable potential for biomedical engineering applications such as bone graft and implant.

## Background

Worldwide, there is an estimated amount of 56 million bone fractures due to an increased life expectancy and thus the occurrence of age-related illnesses such as osteoporosis, a disease, which leads to a loss of bone mass [1]. In the world, the lifetime risk of a bone fracture is 40–50 % for women and 13–22 % for men [2]. Since life expectancy is on the rise all over the world, the frequency of such medical issues will only grow further. Hence, an expansion of the market for biomaterials that can be used in bone tissue engineering is to be expected. The financial impact of bone fractures is substantial; in the US alone the costs of bone fracture treatments for the health care system were situated in the range $12.2–$17.9 billion in 2002 [3]. A reduction of such costs by finding new highly reliable but low-priced bone substitute materials has great potential benefits for society.

Today’s state-of-the-art bone substitute is synthetic hydroxyapatite [HA, Ca_10_(Po_4_)_6_(OH)_2_)] [4]. Its composition is similar to the mineral constituent of bones or teeth [5]. HA can be produced synthetically by various chemical routes such as: precipitation, hydrothermal method, hydrolysis of other calcium phosphates or sol–gel process [6].

However, HA can be also derived from bovine femur bones and it is already available as a commercial product under the Cerabone^®^ and Endobon^®^ trademarks [7].

Bovine femur bones show a micro-hardness which ranges between 47.92 ± 3.98 HV and 71.99 ± 8.97 HV. Bone is a nanocomposite constituted of HA nanocrystals (bone mineral sums up to ~70 wt% of the bone mass, the rest being collagen and other small amounts of proteins and inorganic salts) embedded in a matrix of collagen microfibrils [8]. The bone hardness values differ with the orientation of HA crystallites-collagen microfibrils assembly. The more crystals are perpendicular to the measuring surface, the harder is [9]. Human bone has a compressive strength of ~200 MPa [10].

Unlike bone, pure HA is a material with weak mechanical properties. The compressive strength ranges from 12 MPa (when sintered at 1000 °C and previously compressed at 350 MPa) to 64 MPa (at 1200 °C, 350 MPa). The micro-hardness ranges from 85 HV (at 1000 °C, 350 MPa) to 170 HV (at 1200 °C, 350 MPa) [11]. Other sources report a micro-hardness ranging from 61 HV (when sintered at 1100 °C and previously compressed at 156 MPa) to 200 HV (at 1300 °C, 156 MPa) [12], and a compressive strength around 50 MPa (for 1200 and 1300 °C sintering temperatures) [13].

One method to improve the mechanical properties of HA is the incorporation of glass–ceramics derived from silica—or phosphate-based bioactive glasses. The result is a composite material with higher strength and improved biocompatibility [14]. For example, the compressive strength of HA admixed with 2.5 wt% Na_2_O–CaO–P_2_O_5_ glass, sintered at 1200 °C, is 110 MPa [15].

In this study, natural pumice (NP)—a naturally occurring glass–ceramic of volcanic origin—was used to reinforce bovine-derived hydroxyapatite (BHA). NP is the product of lava being cooled and depressurised, e.g. when a volcanic eruption takes place. It occurs only when enough gases, such as H_2_O, CO_2_ and SO_2_, are dispersed in magma. The liquid magma melt can then transform into single fragments of foamy melt within the gas phase. This process is called magmatic fragmentation [16, 17]. It typically happens during plinian volcanic eruptions, which are characterised by a catapulting of mostly coarse particles (>1 mm) into the air using them in a relatively large area (ca. 1500 km^2^) around the volcano [18, 19]. NP is a highly porous material with a porosity of about 90 %, with all the porosity being interconnected. The pores are often of cylindrical shape, since the maximum packing density of a cylinder is higher than those of a sphere [20]. The density of NP is 1.2–1.4 g/cm^3^ and its compressive strength is 1.72 ± 0.12 MPa [21]. It is composed of mainly SiO_2_ (60–75 wt%), Al_2_O_3_ (13–17 wt%), Na_2_O–K_2_O (7–8 wt%) and smaller amounts of Fe_2_O_3_, TiO_2_ and CaO, etc. [21].

In-vitro testing of NP has shown good MG63 cell viability [19]. As aforementioned, BHA is already in use for medical applications [22], but it offers only limited mechanical properties [23]. However, previous studies with composites of HA and SiO_2_-based bioglass have shown promising results regarding both biocompatibility and mechanical strength [22, 24]. For example, HA has been reinforced with the SiO_2_-based 45S5 Bioglass^®^ and sintered by using a heat-treatment program with a maximum temperature of 1000 °C, resulting in a higher strength [24]. On the other hand, there are also studies that state that the addition of 2 wt% SiO_2_ to HA does not change the compressive strength [25], therefore it is suggested that the other alkali and alkali-earth oxides typical of bioglass formulations might play the prominent role in the improvement of the mechanical response. However, in vitro tests in simulated body fluids and osteogenic cell cultures demonstrated that a composite of HA with a silicate-based additive has greater bioactivity and biocompatibility than pure HA [25].

With all this in mind, it stands to reason to investigate a new composite material fabricated from sustainable resources such as natural pumice and bone derived hydroxyapatite. The aim of this study is to introduce a new bio-inspired composite (NP-BHA) eliciting biocompatibility and mechanically superior properties, thus suitable for bone reconstruction applications.

## Methods

### Materials

Naturally occurring pumice was collected from the Isparta province located in south Turkey. The powder composition, as estimated by energy dispersive spectroscopy, is shown in Table 1. HA was fabricated from commercially available bovine bones (CarrefourSA, Istanbul, Turkey), using as preparation recipe the technological protocol described elsewhere [11]. The experimental procedure for the fabrication of the BHA powder was conducted in accordance with the European Regulation No. 722/2012 and ISO 22442/2007 standard.

**Table 1:** Composition of the natural pumice used in our experiments [41]

SiO_2_ (%)	Al_2_O_3_ (%)	Fe_2_O_3_ (%)	MgO (%)	CaO (%)	Na_2_O (%)	K_2_O (%)	TiO_2_ (%)	SO_3_ (%)	Other (%)
68	16.86	2.72	0.65	1.2	3.58	5.71	0.34	0.12	0.86

### Preparation of NP-BHA

The natural pumice and bovine hydroxyapatite powders were grounded and sieved separately to achieve a mean grain size of ~100 µm. The HA powder was then mixed with 5 and 10 wt% of NP powder, respectively. The samples will be further denominated as 5 wt% NP-BHA and 10 wt% NP-BHA. Each mixture was then further grounded for 4 h in the ball mill. The samples were then prepared according to British standard 7253: The mixtures were compressed between two hardened steel bodies at 350 MPa. The resulting cylinders were then sintered at different temperatures (1000, 1100, 1200 and 1300 °C) for 4 h using a HT 16/17 furnace (Nabertherm GmbH, Lilienthal, Germany). The procedure used is according to previous research conducted by Gunduz et al. [11].

### NP-BHA composite characterization

The morphology of the samples prior and after the in vitro testing was investigated by scanning electron microscopy (SEM), using a SEM JEOL 590, Tokyo, Japan. The bonding configuration and identification of functional groups was performed by Fourier Transform Infrared (FTIR) spectroscopy, using a Perkin Elmer Spectrum BX apparatus in attenuated total reflectance mode (1.8 diameter Pike-MIRacle Diamond Head). The spectra were collected over a range of 1800–550 cm^−1^ by recording 128 individual scans at a resolution of 4 cm^−1^. The identification of crystalline phases was made by X-ray Diffraction (XRD) using a Bruker D8 Advance diffractometer, with Cu K_α_ (λ = 1.5418 Å) radiation, equipped with a high efficiency linear detector of Lynx Eye type. The samples were measured in symmetric geometry in the angular range 2θ = 5°–60°, with 0.04° step size and 10 s acquisition time per step.

### Mechanical testing

The compression test was conducted with a speed of 2 mm/min using the universal tensile testing machine DVT (Devotrans Inc., Istanbul, Turkey). The micro-hardness (HV) was tested with a load of 200 g and 20 s dwell time using the HMV-2 (Shimadzu, Kyoto, Japan). This procedure was carried out which are performed according to previous research [11].

### Cytocompatibility assays

Cytocompatibility of the samples (NP-BHA composites, as well as simple NP and BHA controls) was evaluated by culturing with primary human osteosarcoma cells (Saos-2) provided by the American Type Culture Collection (ATTC). The osteoblast-like Saos-2 cell line is widely used as an in vitro biocompatibility model for materials with bone regeneration applications [38, 39]. The cells were routinely grown in Dulbecco’s Modified Eagle Medium (DMEM) medium (Sigma Chemical, St. Louis, MO, USA) supplemented with 10 % volume fraction of calf serum (Gibco, Rockville, MD, USA), 100 UI/ml penicillin, 100 µg/ml streptomycin, and l-glutamine. Cells were subcultured once a week using trypsin and maintained at 37 °C in an incubator with humidified 5 % CO_2_ atmosphere. The medium was changed every 3 days. The confluent cells were used in cytotoxicity tests [26].

First, the conditioned medium was prepared to understand any possible toxic effect induced by possible ionic leach-out product from the samples into the medium. For this aim, 10 mL fresh medium was added in tubes with 1 g of tested material (NP-BHA composites, NP and BHA), which were kept in the incubator. One week after the conditioned medium was extracted, and later used in cytotoxicity tests.

The Saos-2 cells (10^5^ cells per well) were seeded in 98-wells micro plate, conditioned medium and unconditioned DMEM medium as control, Plates were transferred to a humidified atmosphere incubator, and after 3 days the cell proliferation was measured by classic MTT test. The culture media were removed, and 10 uL (5 mg/ml) of MTT (Sigma Chemical, St. Louis, MO, USA) solution was added to each well. Following incubation at 37 **°**C for 4 h in a humidified 5 % CO_**2**_ atmosphere, the media was discarded. The precipitated formazan was dissolved in dimethyl sulfoxide (150 µL per well), and optical density was evaluated using a micro plate spectrophotometer at a wavelength of 570 nm.

The cytotoxicity assays were conducted in triplicate. A t test was performed to determine the statistical significance between experimental groups. Kruskal–Wallis test was used to compare the groups. A value of p <0.05 was considered to be statistically significant.

Each specimen (5.0 mm in diameter and 1.0 mm in length) was sterilized with 96 % ethanol and ultraviolet radiation. After washing the samples with medium, they were put in wells with 5 mL of fresh medium (without cells), and kept overnight in incubator. Saos-2 cells were seeded on the surface of pre-wetted specimens (10 × 10^5^ cells per specimen). The specimens were then placed in 6-wells cell culture plates, and maintained for 3 h in incubator to allow the cells to attach. Then, additional 1 mL culture medium was added into each well, and transferred into the incubator. The medium was changed at 2 days. After 4 days, the media were removed, and the cells were fixed with 3 % volume fraction of glutaraldehyde, subjected to graded alcohol dehydration, rinsed with isoamyl acetate, and observed by a scanning electron microscopy.

### Antimicrobial activity evaluation

First, antimicrobial activity of NP and NP-BHA samples was determined by using a qualitative diffusion disk test method [27]. *Enterococcus faecalis* ATCC 29212, Staphylococcus aureus ATCC 6538 and Escherichia coli ATCC 25922 strains were used as test microorganisms in this study. The test strains (10^5^ CFU/mL) were inoculated on MHA (Muller Hinton Agar) surface using Drigalsky loop. The NP and NP-BHA pellets of 10 mm in diameter were positioned at the centre of the Petri plates. The plates were incubated at 37 °C up to 24 h. The microbial inhibition zones on the samples’ surface were evaluated at 6 and 24 h.

A quantitative analysis for the confirmation of antimicrobial activity of NP and NP reinforced materials against *E. faecalis* ATCC 29212 strain has been performed. Powdered specimens were added to 9 mL Muller Hinton broth to final concentration of 10 mg/mL. *Enterococcus faecalis* test strain (10^4^–10^5^ CFU/mL) was added and specimens incubated at 37 °C for 24 h. 1 mL sample was taken from this mixture and analysed by spectrophotometry for microbial counts at the 0, 6 and 24 h. The analyses were performed in triplicate.

## Results and discussion

**Fig. 1:**
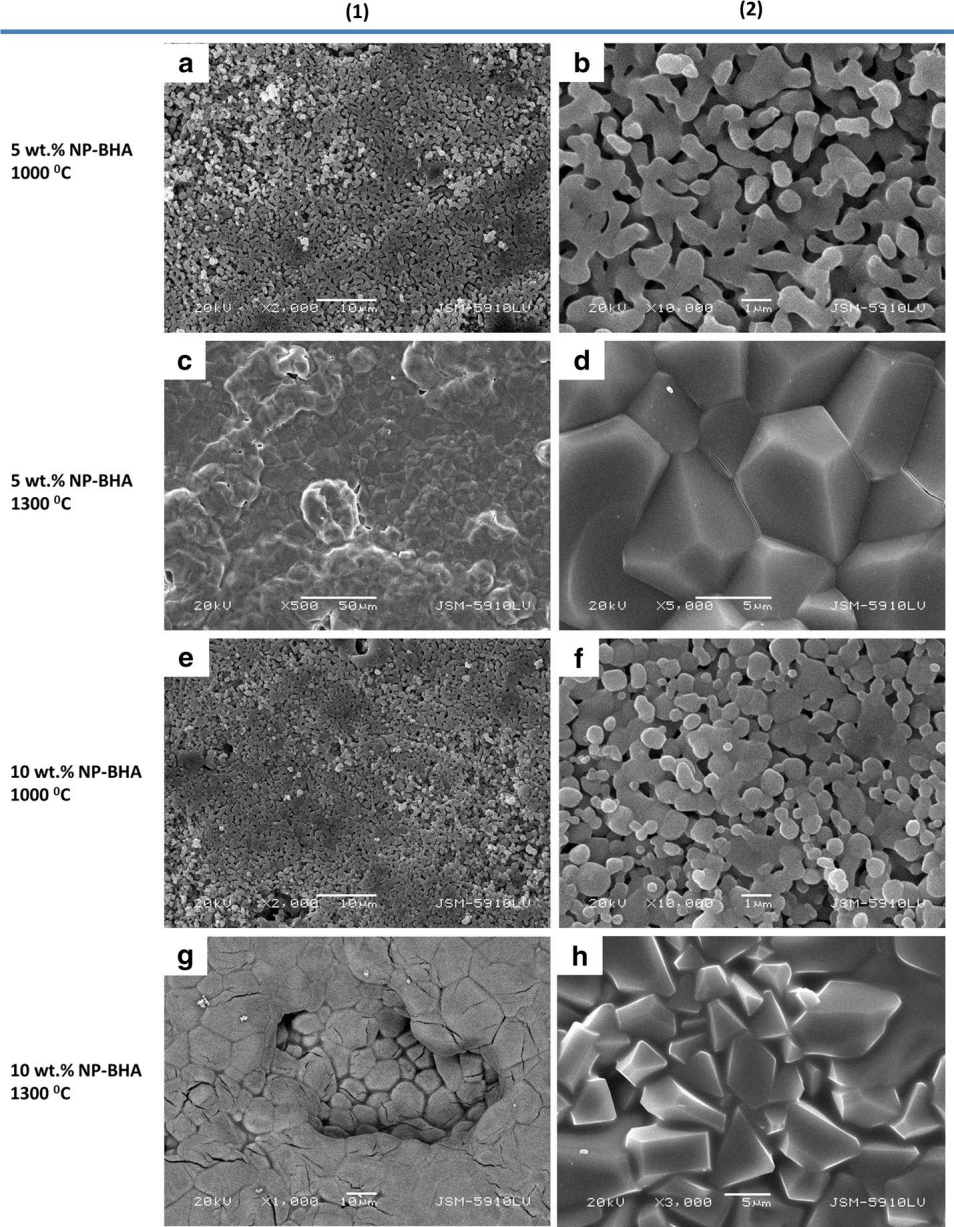
SEM images recorded on the surface of the 5 wt% NP-BHA (**a**–**d**) and 10 wt% NP-BHA cylinders sintered at 1000 °C (**a**–**f**) and 1300 °C (**c**, **d**, **g**, **h**)

The 5 wt% NP-BHA composite sintered at 1000 °C has a porous surface with sponge-like appearance, formed by grains sutured by necks (Fig. 1a). Scarce needle-shaped crystal precipitates were also noticed (Fig. 1b). Figure 1c illustrates the same composite sintered at 1300 °C. The structure looks obviously radically different, having a more homogeneous surface (Fig. 1c) composed of closely-packed grains with regular polyhedral shapes (hexagonal-like) and well-defined grain boundaries instead of pores (Fig. 1d). Figure 1e, f, g and h shows the morphology of the 10 wt% NP-BHA composite sintered at 1000 and 1300 °C, respectively. The 10 wt% NP-BHA samples sintered at 1000 °C had also a porous structure (Fig. 1e), with occasional crystals (Fig. 1f), but they are not needle-shaped anymore. Also the sizes of the pores seem to be smaller with respect to the 5 wt% NP-BHA, whilst and the grains are connected by wider necks, leading to an overall lower porosity fraction. The 10 wt% NP-BHA samples sintered at 1300 °C showed an obviously different morphology, defined by the absence of any pores and the presence of well-developed grains having rather various polyhedral shapes and sizes (Fig. 1h). Some grains appear to be cracked (Fig. 1g). The cracks could be related to the allotropic transformation of β-TCP into α-TCP [28].

**Fig. 2:**
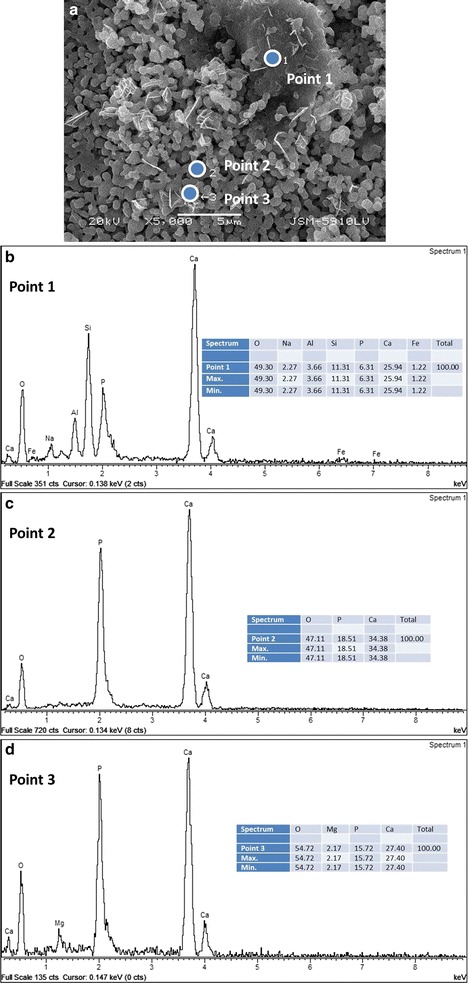
**a** SEM image collected at a magnification of 5000× in the case of the 5 wt% NP-BHA cylinder sintered at 1000 °C. (**b**, **c**, **d**) EDS analyses (qualitative—spectrum and quantitative—table in inset) for the regions indicated in (**a**): point 1 (**b**), point 2 (**c**), and point 3 (**d**)

The SEM image displayed in Fig. 2a. The regions where EDS measurements have been carried out in the case of the 5 wt % NP-BHA sample sintered at 1000 °C. Point 1 is located on a homogeneous outgrowth, point 2 is situated in the middle of the porous part of the surface, and point 3 was chosen in a region rich in needle-shaped crystals. The EDS spectra along with the quantitative results are presented in Figs. 2b–d. One can notice that different sample compositions are obtained for the three regions indicated in Fig. 2a. Region 1 is composed of mainly oxygen (49 at.%), calcium (26 at.%) silicon (11 at.%) and smaller amounts of phosphorous, aluminium, sodium and iron (see Fig. 2b). The porous region (2) is exclusively composed of oxygen (47 at.%), calcium (34 at.%) and phosphorous (19 at.%) (Fig. 2c). The area rich in needle-shaped crystals (3) had a similar composition to region 2, with oxygen (55 at.%), calcium (27 at.%), phosphorous (16 at.%), but also a low amount of magnesium (2 at.%) (Fig. 2d).

**Fig. 3:**
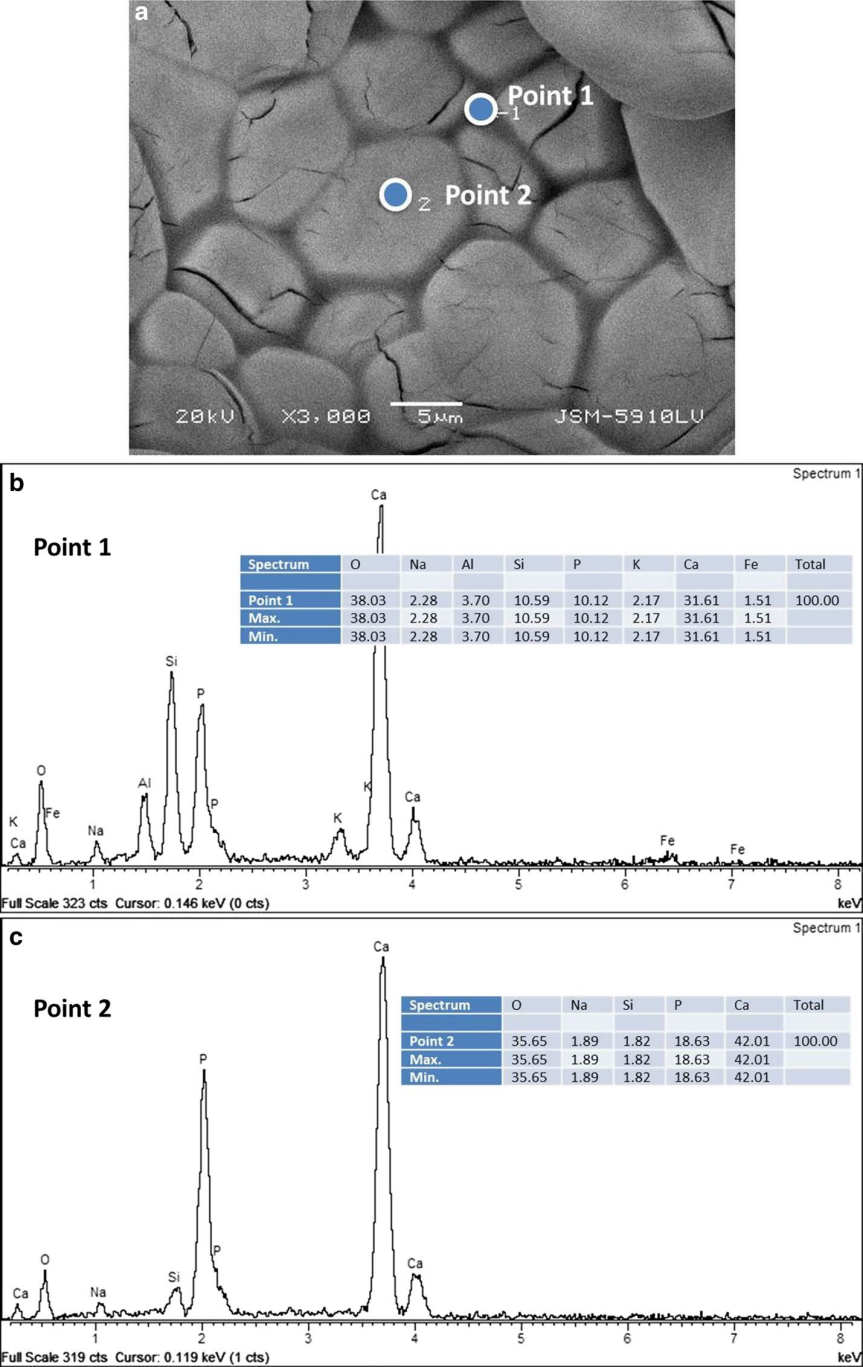
**a** SEM image collected at a magnification of 3000× in the case of the 10 wt% NP-BHA cylinder sintered at 1300 °C. **b**, **c** EDS analyses (qualitative—spectrum and quantitative—table in inset) for the regions indicated in (**a**): point 1 (**b**) and point 2 (**c**)

The SEM image in Fig. 3a shows the regions where EDS measurement have been performed for the 10 wt% NP-BHA composite sample sintered at 1300 °C. Point 1 is located on a grain boundary and point 2 is situated in the centre of a grain (Fig. 3a). On the grain boundary region, the composition is mainly consists of oxygen (38 at.%), calcium (32 at.%), silicon (11 at.%) and phosphorous (10 at. %), as well as smaller amounts of aluminium, sodium, potassium and iron (Fig. 3b). The grains have as main constituents, which are calcium (42 at.%), oxygen (36 at. %) and phosphorous (19 at.%), and smaller amounts of sodium and silicon (Fig. 3c).

**Fig. 4:**
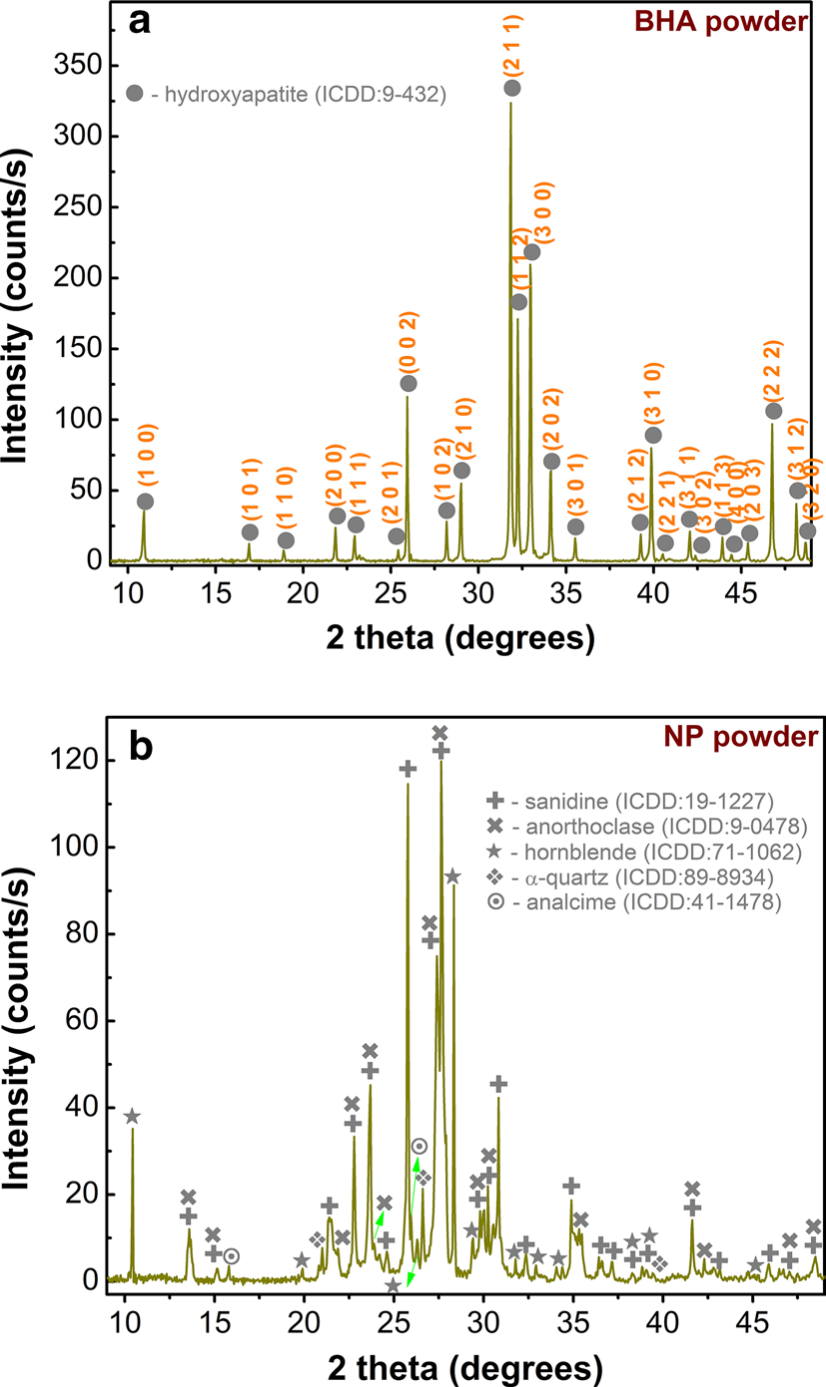
XRD patterns of raw starting materials: **a** BHA and **b** NP powders

The XRD measurements have been performed first on the starting materials: the bovine hydroxyapatite and natural pumice powders (Fig. 4). One can notice the single-phase nature of the BHA material, consisting of an isotropic highly crystalline hydroxyapatite (ICDD: 00-009-0432) (Fig. 4a). On the other hand, the analysis of the natural pumice powder revealed sanidine (K,Na)Si_3_AlO_8_ (ICDD: 00-019-1227) and/or anorthoclase (Na,K)Si_3_AlO_8_ (ICDD: 00-009-0478) as main crystalline phases, along other minor signals assigned to hornblende (ICDD:01-071-1062), α-quartz (ICDD: 01-089-8934) and analcime Na(Si_2_Al)O_6_•H_2_O (ICDD: 00-041-1478) (Fig. 4b). The juxtaposition of the diffraction lines of sanidine and anorthoclase kindred phases make a safe association difficult. However, we note that previous studies have reported sanidine as an ubiquitous phase in NP of various origins [29].

**Fig. 5:**
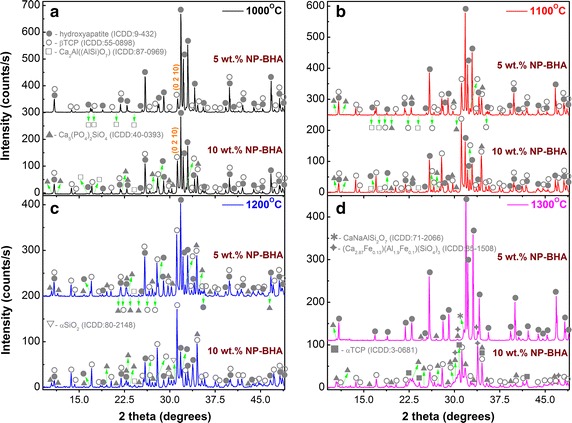
XRD patterns of NP-BHA composites sintered at: **a** 1000 °C; **b** 1100 °C; **c** 1200 °C; and **d** 1300 °C

The structural evolution of the NP-BHA composites with sintering temperature is presented in Fig. 5. After sintering at 1000 °C, the 5 wt% NP-BHA composite consisted mainly of hydroxyapatite, with β-tricalcium phosphate (β-TCP, whitlockite) (ICDD: 00-055-0898) as a secondary phase (Fig. 5a). Shallow peaks assigned to a Ca_2_Al(AlSi)O_7_ type phase (ICDD: 01-087-0969) have been also identified. The thermal decomposition of BHA to β-TCP is known to start sintering at temperatures as low as 800–850 °C We emphasize that the presence of β-TCP in such biomaterials is known to play an osteoconduction boosting role due to its higher resorption rate [30]. BHA—β-TCP mix materials (also called biphasic calcium phosphates) are under worldwide focus, in the search of solutions which can harmoniously match the rate of bioceramic resorption with the rate of new bone formation. The presence of the dicalcium aluminium aluminosilicate phase indicates the occurrence of a reaction between the matrix material (BHA) and the reinforcing phase (NP). In the case of 10 wt% NP-BHA composite sintered at 1000 °C, an increase of the β-TCP content is noticed on the expense of BHA, along with more conspicuous peaks of the minoritary Ca_2_Al(AlSi)O_7_ and the appearance of a new crystalline NP-BHA mix phase: Ca_5_(PO_4_)_2_SiO_4_ (ICDD: 00-040-0393).

The sintering treatment performed at 1100 °C hinted towards a continuous transformation of HA into β-TCP with temperature (Fig. 5b), and also induced the increase in crystallinity of the Ca_2_Al(AlSi)O_7_ and Ca_5_(PO_4_)_2_SiO_4_ phases. If in the case of 5 wt% NP-BHA sample, BHA is still the prominent constituent, whilst the 10 wt% NP-BHA material is now dominated by β-TCP, with HA as a secondary phase.

After the 1200 °C sintering, the β-TCP content increases for both 5 and 10 wt% NP-BHA composites (Fig. 5c). If in the case of 5 wt% NP-BHA, the Ca_5_(PO_4_)_2_SiO_4_ reaches its zenith in terms of crystallinity, in the case of 10 wt% NP-BHA the calcium phosphate silicate phase peaks seem less prominent and the emergence of a new phase—α-SiO_2_ (ICDD: 01-080-2148)—is now detected.

The sintering treatment performed at 1300 °C induced radical structural modifications (Fig. 5d). Interestingly, the diffraction pattern of the 5 wt% NP-BHA sample indicated only the presence of hydroxyapatite along two new minor phases: CaNaAlSi_2_O_7_ (ICDD: 01-071-2066) and (Ca_2.87_Fe_0.13_)(Al_1.9_Fe_0.1_)(SiO_4_)_3_ (ICDD:01-085-1508). The β-TCP and Ca_2_Al(AlSi)O_7_ phase are now absent, whilst the intensity of the Ca_5_(PO_4_)_2_SiO_4_ diffraction lines diminished in comparison with the 5 wt% NP-BHA sample sintered at 1200 °C. On the other hand, the 10 wt% NP-BHA diffractogram indicated the total decomposition of BHA and the partial transformation of the low-temperature polymorph β-TCP into the high-temperature polymorph α-TCP (ICDD: 00-003-0681). Also, after the sintering treatment performed at 1300 °C, the diffraction lines of 10 wt% NP-BHA are broader, whilst their intensity is significantly reduced. The presence of Ca_5_(PO_4_)_2_SiO_4_ is still noticeable, along the newly emerged (Ca_2.87_Fe_0.13_)(Al_1.9_Fe_0.1_)(SiO_4_)_3_ phase.

The thermal decomposition of BHA is accompanied by an overall decrease in intensity of the diffraction lines and their broadening. The regular line shifts recorded for both BHA and β-TCP phases during sintering, with respect to the reference ICDD file positions, advocate for the occurrence of various lattice ionic substitutions with the species appertaining to the reinforcing NP agent.

The XRD results also suggest a dependency of the decomposition rate of BHA on the mass fraction of NP within the composites. One can observe that, in the case of 5 wt% NP-BHA, the BHA transformation into TCP takes place at a slower pace than in the case of 10 wt% NP-BHA. The driving force of BHA decomposition is given by its gradual dehydration with increasing sintering temperature [31]. After the release of water molecules from the HA structure, a dehydrated unstable and faulty lattice is created, which is highly susceptible to both chemical reactions, ionic substitutions from surrounding media, and thereby decomposition or nucleation of new phases. In such a case, the amount of reinforcing compound in the composite will play a decisive role on the BHA decomposition speed at a given temperature, as the hydroxyl loses during sintering can be compensated by various chemical species of pumice found in intimate contact with BHA. The different thermal expansion coefficients of BHA and NP crystalline phases can also induce high stresses at the grain boundaries and enhance the thermal decomposition of the BHA phase [32].

The TCP conversion to BHA at elevated temperatures is not unprecedented [33]. Kong et al. have stated that at high temperatures the Ca ions from the HA regions have the ability to diffuse into the β-TCP regions and convert the TCP into a Ca-deficient HA [33].

Our results seem to indicate that such a phenomenon can occur only if a larger concentration of BHA should pre-exist in a given sample, such as the case of 5 wt% NP-BHA (Fig. 5c vs. d). If the β-TCP is the dominant phase in the composite, the sintering will lead to a further decomposition of BHA, and eventually to the β-TCP transformation into its high-temperature polymorph, α-TCP (Fig. 5c vs. d).

**Fig. 6:**
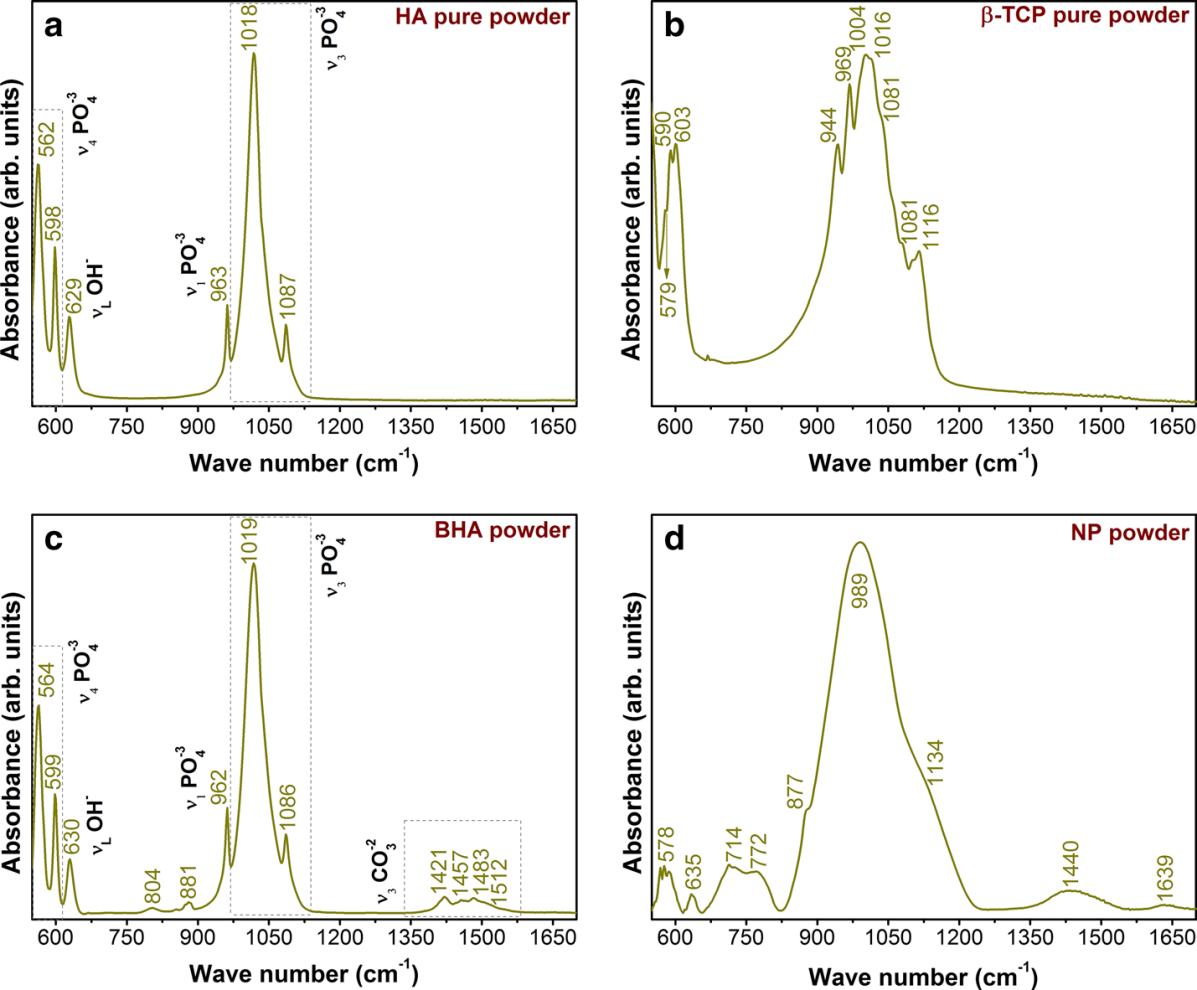
Comparative FTIR spectra of pure hydroxyapatite (**a**) and β-tricalcium phosphate (**b**) powders and raw starting materials: BHA (**c**) and NP (**d**) powders

Figure 6 presents the FTIR spectra of the raw starting materials (c,d) in comparison with two control samples: pure hydroxyapatite (Sigma-Aldrich) and β-tricalcium phosphate (Sigma-Aldrich) powders. The BHA material exhibited (Fig. 6c) all the characteristic vibration bands of hydroxyapatite (Fig. 6a) due to the phosphate functional groups (prominent ν_3_ asymmetric stretching mode positioned at 1019 and 1087 cm^−1^, the ν_1_ symmetric stretching centred at 962 cm^−1^, and the ν_4_ bending modes peaking at 564 and 599 cm^−1^) and the structural hydroxyl units (librational OH^−^ mode situated at 630 cm^−1^) The sharp allure of the IR absorption bands indicated the high crystalline nature of the BHA material in perfect agreement with the aforementioned XRD results (Fig. 4a). The slight shifts of the IR bands of BHA with respect to the pure BHA suggested short-range order structural alterations due to stoichiometry modifications typical to the bone mineral [34]. In fact the bone mineral is a non-stoichiometric carbonated hydroxyapatite, enclosing important amounts of ionic species such as (CO_3_)^−2^ (3–8 wt%), Mg^2+^ (~0.5 wt%), and Na^+^ (~0.7 wt%), along other trace elements (K, Si, Cl, F, S, Fe, Cu, Ni, Sr, Zn) [35].

Few other additional vibration bands, with maxima at 804, 881, 1421, 1457, 1483, and 1512 cm^−1^, can be noticed in the BHA powder spectrum (Fig. 6c), which emphasize the biological origin of this material. The presence of absorption bands at 881, 1421, 1457, 1483, and 1512 cm^−1^ is attributed to the ν_2_ bending and ν_3_ stretching modes of carbonate (CO_3_)^−2^ groups, and indicate a AB-type double substitution into the HA lattice: (i) substitution of OH^−^ with (CO_3_)^−2^ (A-type substitution) and ii) substitution of (PO_4_)^−3^ with (CO_3_)^−2^ (B-type substitution) due to charge compensation necessity The weak band at 881 cm^−1^ can be also the result of the overlapping of the vibration band of (HPO_4_)^−2^ with the ν_2_ bending mode of carbonate The existence in bone mineral of ions in non-apatitic domains has been documented in the past and it is usually located in a hydrated layer on the surfaces of the apatitic crystals Such a layer is known to contain prevalently bivalent ions such as HPO_4_)^−2^ and (CO_3_)^−2^ The peak at 804 cm^−1^ advent from the OH^−^ deformation band of BHA structure [36].

The IR spectrum of NP is dominated by a main broad peak centred at 989 cm^−1^, having two additional side shoulders positioned at 877 and 1134 cm^−1^ (Fig. 6d). The bands can be assigned to the characteristic vibrations of silicate groups. The bands’ broadening is consistent with a less-ordered structure. The band at 1134 cm^−1^ can be ascribed to the ν_3_ asymmetric stretching vibration of Si–O–Si bonds The band centred at 989 cm^−1^ evidences by the asymmetric stretching vibrations of Si–O–Al bonds The faint band peaking at 877 cm^−1^ can be attributed to the SiO_4_ units with non-bridging oxygen atoms (most probably Q^3^ and Q^2^ units) [37]. The other vibrational bands located on the low wave numbers region unveiled the typical symmetric stretching (714, 772 cm^−1^), bending (635 cm^−1^) and rocking (578 cm^−1^) vibrational modes of various silicate groups The shift of IR bands to low wavenumbers is generated by incorporation of Al^3+^ into the Si^4+^ sites and the structural modifications determined by the breaking of oxygen bridges due to the presence of the alkali and alkali-earth species, and consequently their charge compensation necessity. Such phenomena are prone to take place as the excess negative charge arising from the Al^3+^→ Si^4+^ replacement might not be enough to balance the positive ions (alkali and alkali-earth cations) charge, which are presented in NP and sum up to ~11 wt% of its composition.

The Q^2^, Q^1^, and Q^0^ phosphate units yield IR vibration bands in the 1400–400 cm^−1^ range However, the vibrations of phosphate groups present in NP are difficult to be emphasized because of the low P content of the material and the superimposition of prominent of silicate units in the fingerprint absorption region. The presence of the ν_3_ asymmetric stretching lines of (CO_3_)^−2^ (~1440 cm^−1^) and bending vibrations of adsorbed water molecules (1639 cm^−1^) were also evidenced. We stress that the 877 cm^−1^ peak can be associated only partially to the ν_2_ bending of carbonate, as the intensity of such a vibration mode is considered to be only ~1/5 of the ν_3_ asymmetric stretching band of carbonate [35].

**Fig. 7:**
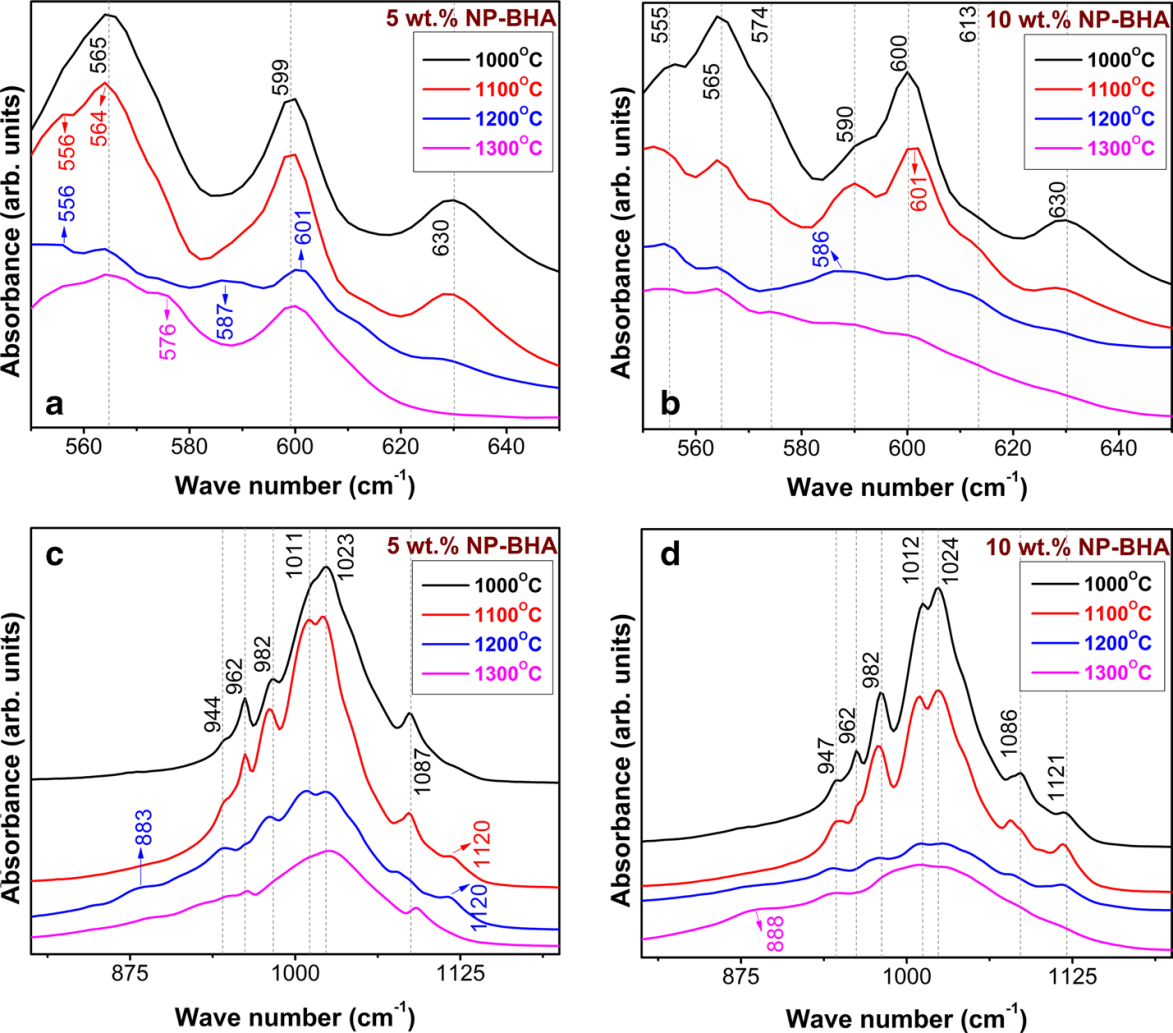
FTIR spectra of 5 wt% NP-BHA (**a**, **c**) and 10 wt% NP-BHA (**b**, **d**) composites sintered at different temperatures, in two spectral regions : 550–650 cm^−1^ (**a**, **b**) and 800–1200 cm^−1^ (**c**, **d**)

The short-range structural evolution of the NP-BHA composites with the sintering temperature is depicted in Fig. 7. For a better observation of the events, the FTIR graphical representations have been separated in two spectral regions: 650–550 cm^−1^ (Fig. 7a, b) and 1200–800 cm^−1^ (Fig. 7c, d). We decided to follow only the fingerprint IR region, as at temperatures higher than 800 °C, carbonate, adsorbed water and other contaminants or organic residues are already eliminated [38].

Both low (Fig. 7a, b) and high (Fig. 7c, d) wavenumbers regions of the composites are dominated by vibrational bands of phosphate functional groups. The more intricate IR envelops of 10 wt% NP-BHA composite with respect to the one of 5 wt% NP-BHA sample, at every sintering temperature, support the higher rate and degree of BHA decomposition. Due to the aforementioned coexistence in the same spectral regions of the phosphate and silicate vibrational mode, the NP bands are more difficult to be unravelled.

The triply degenerated bands of ν_3_ asymmetric stretching mode (1011/1012, 1023/1024 and 1087/1086 cm^−1^), and of the ν_4_ bending mode (556/555, 565/565 and 599/600 cm^−1^), as well as of the ν_1_ symmetric stretching (962/962 cm^−1^), evidenced in the 5 and 10 wt% NP-BHA composites, belong to the structural vibrations of phosphates groups in both BHA and β-TCP (Fig. 7a, b).

One can notice that the libration band of structural OH^−^ units of BHA (Fig. 7a, c vs. a, b) is reducing progressively with the increase of sintering temperature. The process is more acute in the case of 10 wt% NP-BHA composite. As stated before (see XRD chapter), the dehydration of HA plays a prominent role in its decomposition kinetics. As a consequence the phosphate functional groups enter a restructuring process, which is clearly illustrated by FTIR spectra (Fig. 7).

The bands positioned at 944/947 and 982/982 cm^−1^ are distinctive to a β-TCP phase and are present for both composites after the sintering treatment at 1000 °C. The 1121 cm^−1^ shoulder is present in the 10 wt% NP-BHA (Fig. 7d) after the 1000 °C sintering, and appears (at 1120 cm^−1^) for 5 wt% NP-BHA (Fig. 7c) only after a sintering treatment performed at a higher temperature (1100 °C), and disappears after the 1300 °C sintering in the case of both types of composites. The band is typical for a β-TCP heated at temperatures ≥1000 °C [39], and its evolution with increasing sintering temperature correlates well with the gradual decrease of the libration band of structural OH^−^ units. Thus, the FTIR results are in good agreement with the XRD findings, which suggested that the BHA lattice dehydroxylation allows for the structuring of the β-TCP counterpart.

The new shallow shoulder emerging at 883/888 cm^−1^ after the sintering at 1200 °C for both composites (Fig. 7c, d) could be ascribed to the increase of short-range order of silicate based compounds, whose presence was confirmed by the XRD results (Fig. 6). After the sintering at 1300 °C, significant modifications were recorded in the IR spectra of NP-BHA composites. The 5 wt% NP-BHA exhibits only the bands of the triply degenerated ν_3_ asymmetric stretching and ν_4_ bending modes together with ones of the ν_1_ symmetric stretching. The absence of the libration band of hydroxyl groups (Fig. 7c) suggests the formation of an oxyapatite compound, in good agreement with the XRD observations (Fig. 5). In the case of the 10 wt% NP-BHA (Fig. 7d) the bands became broaden hinting towards a dramatic structural transformation, whilst the characteristic bands of β-TCP (947, 982 and 1121 cm^−1^) are still present, but their intensity is more reduced.

**Fig. 8:**
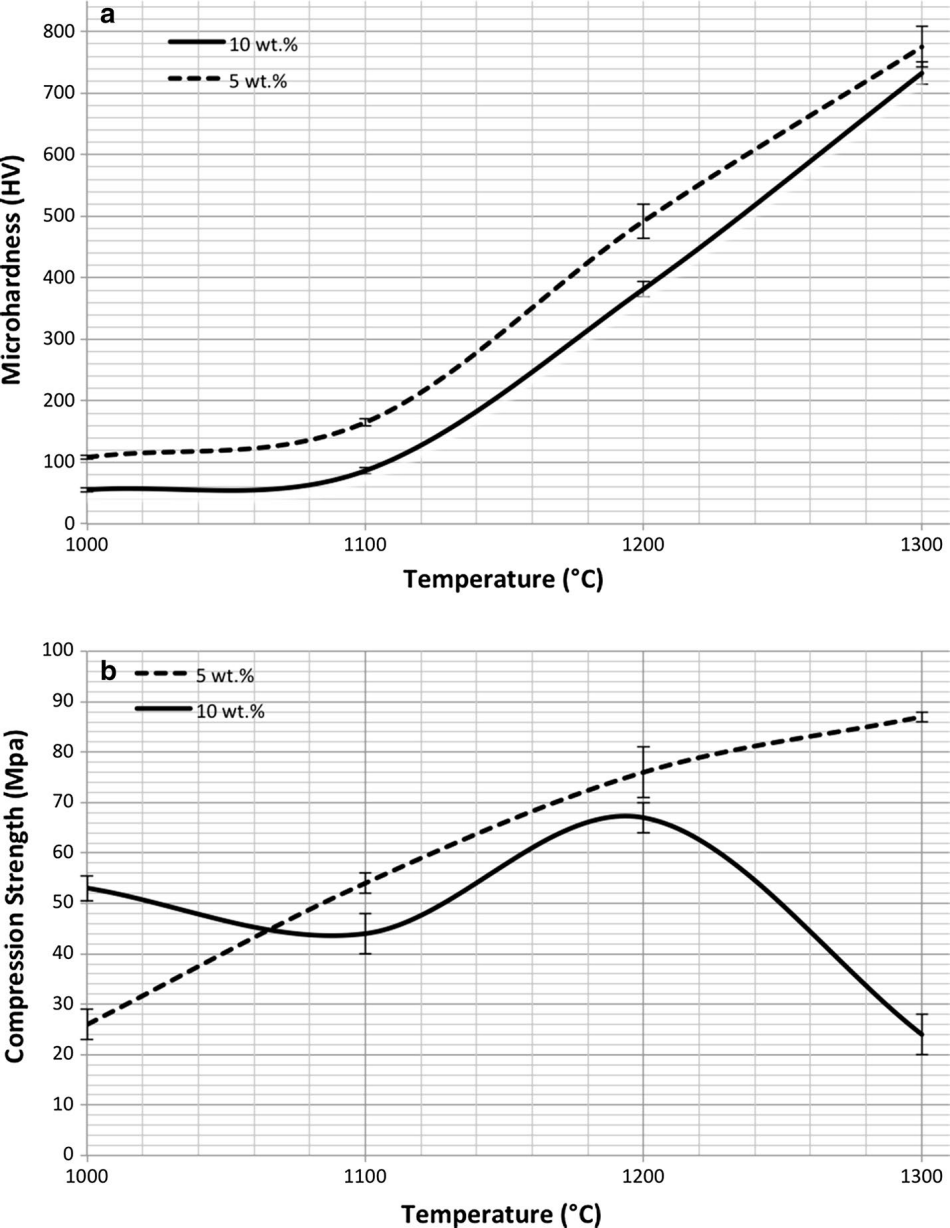
Micro-hardness and (**a**) compression strength (**b**) of BHA reinforced with 5 and 10 wt% NP, sintered at 1000, 1100, 1200 and 1300 °C

The results of the micro-hardness tests performed on the NP-BHA composited sintered at different temperatures are shown in Fig. 8a. The 5 wt% NP-BHA composite is generally harder than the 10 wt% NP-BHA one. Furthermore, the hardness in both composites increases with the sintering temperature. As aforementioned, SiO_2_ and Al_2_O_3_ are the main ingredients of NP (Table 1). Oktar et al. [40] conducted micro-mechanical tests on BHA reinforced with 5 and 10 wt% of SiO_2_ or Al_2_O_3_. In both cases, they observed that hardness decreases when the amount of SiO_2_ or Al_2_O_3_ was raised. Indeed, in our case the 5 wt% NP-BHA composite sample sintered at 1300 °C provided the highest hardness value (775 ± 33 HV). This is an improvement compared to pure HA which has a micro-hardness values in the range 85–200 HV or to bovine femur bones with a micro hardness of 72–148 HV. However, there are commercially available products such as Norian SRS^®^, which possess a micro-hardness of 1326 HV at 200 g load [10], thus higher than that of the NP-BHA composite materials studied herein. But, the NP-BHA production costs would be significantly lower, as it would be based on materials derived from cheap sustainable resources. Gunduz et al. [41] has reported the synthesis of biocomposites based on the addition of inert glass (CIG) addition to BHA. The best microhardness value found for CIG–BHA composites was of 507 HV, which is noticeably lower than that of the 5 wt% NP-BHA composite sample.

Figure 8b the compressive strength values recorded in the case of NP-BHA composites. For sintering temperatures above 1100 °C, the 5 wt% NP-BHA composite elicited higher compressive strength values. Only when applying a sintering temperature of 1000 °C, the 10 wt% NP-BHA composite was stronger than the 5 wt% NP-BHA composite, most probably due to more decreased porosity (see Fig. 1). The highest compressive strength (87 ± 0.5 MPa) was recorded for 5 wt% NP-BHA composite sample sintered at 1300 °C. This is an improvement compared to the 12–64 MPa of pure HA which thus demonstrate the positive reinforcing effect of natural pumice.

Compared to common bone substitute materials, such as tricalcium phosphate (compressive strength: 10.95 ± 1.28 MPa) or commercially available products like Calcibon^®^ (35–55 MPa), Norian SRS^®^ (23–55 MPa) and HydroSet^®^ (14–24 MPa) [42] the NP-BHA composite introduced in the present study has a significantly higher compressive strength. However, it does not meet yet the average compressive strength of human cortical bone, which varies between 100–150 MPa [43]. Goller et al. [44] have added reduced quantities of bioglass (5 and 10 wt%) to BHA to prepare composite structures. The best compression result (~83 MPa) was achieved at a sintering temperature of 1200 °C in the case of the 10 wt% bioglass reinforced material. Thus, a direct comparison shows that the 5 wt% NP reinforced BHA material demonstrated a slightly higher compression value (~87 MPa).

**Fig. 9:**
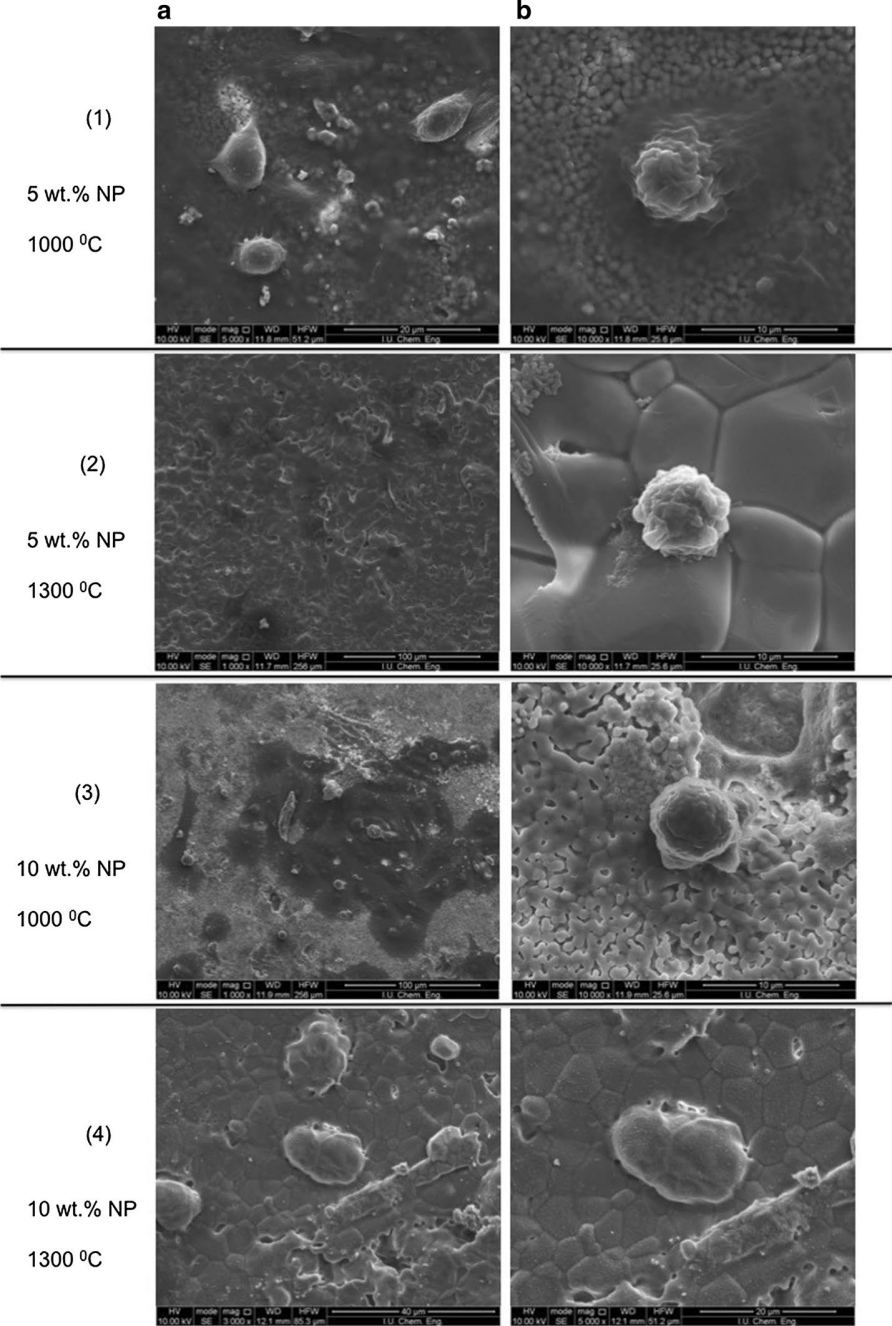
**a** Low and **b** high magnifications SEM images showing of Saos-2 osteosarcoma cells cultured for 4 days on the surface of: (1) 5 wt% NP-BHA as sintered at 1000 °C; (2) 5 wt% NP-BHA as sintered at 1300 °C; (3) 10 wt% NP-BHA as sintered at 1000 °C; and (4) 10 wt% NP-BHA as sintered at 1300 °C

Figure 9 reveals representative SEM images showing Saos-2 cells morphology at 4 days of cell morphology on the 5 and 10 wt% NP-BHA composite sample sintered at 1000–1300 °C. Both the low (a-column) and high magnification (b-column) SEM images indicated that the cells had spread well on the NP-BHA composite surfaces. Quite similar cell morphology was observed on both type of NP-BHA composites sintered at 1000 °C. Interestingly, in the case of these samples, more cells have attached per unit of area, being also much better spread in comparison to the composite samples sintered at 1300 °C. In addition, the Saos-2 cells seem to have attached very well on the composites surface being able to bridge the micropores with their lamellipodia.

**Fig. 10:**
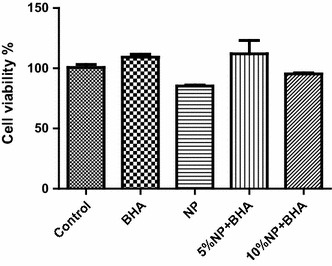
MTT assay histogram. Optical density recorded for the NP, BHA, and NP-BHA composites, expressed in percents with respect to control

The MTT tests (Fig. 10) indicated that the BHA sample and NP-BHA composites had no cytotoxic compared to control. The cell viability was less than that of the control in the case of simple NP, whilst conversely for the pure BHA and 5 wt% NP-BHA was higher, although not statistically significant. Thus, the biocompatibility tests demonstrate that NP-BHA composites have suitable cytocompatibility, and can be recommended for the further development of biomedical applications. A weak microbial inhibition zone at natural pumice (5 wt%) pellet samples for *Enterococcus faecalis* ATCC 29212 strain. The quantitative tests regarding *E.faecalis* microbial development inhibition in liquid media, containing NP and NP-BHA powders strains are shown at Table 2. The results indicate a slight antimicrobial effect of all samples; the greatest effect being detected in the case of NP.

**Table 2:** Antimicrobial activity of natural pumice and NP-BHA composites

Time (h)	NP (CFU/ml)	5 wt% NP-BHA	10 wt% NP-BHA	Control (*E. faecalis*)
0	6.7 × 10^4^	7.4 × 10^4^	7.0 × 10^4^	5.6 × 10^4^
6	8.3 × 10^7^	9.8 × 10^7^	8.3 × 10^7^	1.1 × 10^8^
24	5.0 × 10^8^	1.3 × 10^9^	3.0 × 10^8^	1.8 × 10^9^

## Conclusions

We have successfully synthesized new biocompatible materials, based on cheap sustainable resources [bovine hydroxyapatite (BHA) and natural pumice (NP)], by powder pressing followed by sintering at temperatures in the range of 1000–1300 °C. A synergistic effect, in terms of both cytocompatibility and mechanical performance, has been recorded for the BHA composites reinforced with 5 wt% NP. Their in vitro biocompatibility behaviour was higher than that of pure BHA. Changes in micro-hardness and compression strength can be tailored by the tuning the NP concentration and sintering temperature. NP-BHA composites have demonstrated a remarkable potential for biomedical engineering applications such as bone graft and implant.
